# Consolidation of a WSN and Minimax Method to Rapidly Neutralise Intruders in Strategic Installations

**DOI:** 10.3390/s120303281

**Published:** 2012-03-07

**Authors:** Jesus Conesa-Muñoz, Angela Ribeiro

**Affiliations:** Center for Automation and Robotics, CSIC-UPM, Arganda del Rey, 28500 Madrid, Spain; E-Mail: jesus.conesa@car.upm-csic.es

**Keywords:** intelligent systems, wireless sensor network, decision support systems, minimax algorithm, path planning, risk measure, surveillance system

## Abstract

Due to the sensitive international situation caused by still-recent terrorist attacks, there is a common need to protect the safety of large spaces such as government buildings, airports and power stations. To address this problem, developments in several research fields, such as video and cognitive audio, decision support systems, human interface, computer architecture, communications networks and communications security, should be integrated with the goal of achieving advanced security systems capable of checking all of the specified requirements and spanning the gap that presently exists in the current market. This paper describes the implementation of a decision system for crisis management in infrastructural building security. Specifically, it describes the implementation of a decision system in the management of building intrusions. The positions of the unidentified persons are reported with the help of a Wireless Sensor Network (WSN). The goal is to achieve an intelligent system capable of making the best decision in real time in order to quickly neutralise one or more intruders who threaten strategic installations. It is assumed that the intruders’ behaviour is inferred through sequences of sensors’ activations and their fusion. This article presents a general approach to selecting the optimum operation from the available neutralisation strategies based on a Minimax algorithm. The distances among different scenario elements will be used to measure the risk of the scene, so a path planning technique will be integrated in order to attain a good performance. Different actions to be executed over the elements of the scene such as moving a guard, blocking a door or turning on an alarm will be used to neutralise the crisis. This set of actions executed to stop the crisis is known as the neutralisation strategy. Finally, the system has been tested in simulations of real situations, and the results have been evaluated according to the final state of the intruders. In 86.5% of the cases, the system achieved the capture of the intruders, and in 59.25% of the cases, they were intercepted before they reached their objective.

## 1. Introduction

Still-recent terrorist attacks have revealed a common need to protect the safety of large spaces such as government buildings, airports or power stations. This demand is particularly strong in the European Union and the United States. Nevertheless, the large sizes and crowded spaces of many of these installations require sufficient attention and resources to quickly detect and completely contain an attack. Once a critical situation has been detected, a reaction has to be executed as quickly and accurately as possible in order to efficiently neutralise the crisis. However, the large number of variables required to properly solve the problem (e.g., distances, number of available resources, number of places attacked and blocked doors) involve decision making, which is not always a trivial task.

Surveillance applications have been developed to solve these problems, inspiring significant research in several fields, such as cognitive video and audio [1,2], radio frequency identification (RFID) [3,4], biometric recognition [5], decision support systems [6,7], distributed architectures [8] and sensors network [9,10]. Some approaches combine one or more of these technologies in the context of intrusions. For example, in [11], a system of displaying sensor information in an outdoor environment is presented. In [12], an image processing approach is proposed to detect abnormal behaviours and generate alarms. In [1], a system monitors the interior of a bus with a set of cameras and microphones. The goal of the last system is to recognise suspicious situations by matching sensor information with knowledge stored in a database. In this case, the approach is largely dependent on the database contents. In [13], surveillance is accomplished by recording activities with small, portable technologies, and the proposed approach reports additional information about the behaviours of the subjects recorded. Other authors focus on methods of coordinating distributed sensors in a network [14,15], the goal is to improve the systems’ robustness and capacity for sensing and gathering sensor data, thus speeding up the performance of the network. Raty *et al.* [16] describes a distributed multi-sensor surveillance software system, and [17] presents the proper procedures for correctly testing and validating previous software.

In the former works, a decision-making system is shown, but its limited capabilities only process raw sensor data, sending the information to a security manager interface [6]. Indeed, until now, the responsibility for analysis and decision making has belonged exclusively to the surveillance team, which had no external help in solving the crisis. The team in charge has to process all of the information received in a short time in order to determine the most relevant data and make a decision as quickly as possible to minimise the impact of the attack. The success of the final action depends largely on the speed and accuracy with which the decision was made. It is, therefore, very interesting to consider implementing a system to speed up this task. Consequently, it is imperative to integrate a support system for decision making into the management of infrastructure safety, *i.e.*, a system to assist in the process of evaluating a scenario and recovering a solution to minimise the consequences of an attack and not just a system to detect an abnormal situation. In this context, this paper presents an intelligent system developed into a project that aims to integrate technologies such as video and audio, decision-support systems, human interface, computer architecture, communications networks and communications security among others to achieve an advanced security system (the Hesperia Project [18]). In fact, the system that we describe in this document represents the decision-making core of the security system under development. Therefore, it can make decisions concerning several complex scenarios in real time, *i.e.*, performing the best action among all possible choices while taking into account the most relevant features of the attack. For the recovery of information from the environment and the detection of abnormal situations, the system will rely on a sensors network that is adequately distributed in the protected installation. In this paper, we consider critical intrusion situations, *i.e.*, when someone unidentified is detected and must be neutralised before he reaches important parts of an installation, but anyway, the system could easily be adjusted to respond to any type of attack, as shown below.

The decision system needs a way to detect unidentified presences and to be updated with the most recent information in real time. A location sensors network is used for this purpose. We will describe this network in the following sections. Starting from the positions of the guards and intruders detected by the sensors network, and with the help of a path planner, the intelligent system will choose the best action from among all possible actions.

As previously mentioned, reaction time is an important parameter for adequately managing critical situations. Therefore, a solution to help experts make the right decision in order to increase the speed of intervention would be very valuable. We propose using the Minimax strategy [19], which originated from game theory, as the core of the intelligent system’s decision-making algorithm, adapting it for the intrusion scenario. The aim is to minimise possible losses while maximising potential gain, in this context, this means capturing the intruders as soon as possible, avoiding as much as possible, any damage they can cause to the installations.

Game theory has shown good results in problem solving in many contemporary fields, especially modelling and describing the behaviour of complex systems in economics [20,21]. One of these complex processes is risk analysis in strategic conflict [22]. Some approaches face risk analysis with a Case Based Reasoning system joined to a learning process [6]; others consider it as a game with two players. Still others show how risk analysis can support game theory solutions and how Monte Carlo methods provide insight into optimal game theory solutions in the presence of uncertain payoffs in a terrorist attack scenario [23]. In [24], a novel approach to incomplete information in board games is described. The proposed method is based on the concept of *metaposition*, the merging of a very large set of possible game states into a single entity containing at least every state in the current information set. Some resolution strategies consider surveillance problems as a set of system-adversary interaction problems in which the adversary is modelled as a rational (selfish) agent trying to maximise his utility [25]. Both [26] and [27] focus on the active defence of computer networks with a Markov chain for privilege model to predict attackers’ behaviour and strategies. Interactions between an attacker and a defender are regarded as a two-player, non-cooperative, zero-sum and finite stochastic game. Another example of game strategy application in the intrusion detection context is the Minimax approach, which has been studied with interesting results in [28]. Therefore, we conclude that the two-player approach combined with game strategy presents a suitable method of facing the types of problems considered in this work.

According to these terms and adapting them to our problem, a critical intrusion situation could be viewed as a game with two players whose positions are uncertain (because the sensors work with an approximate estimation and because they will only detect a suspicious person at certain locations limited to their own range of perception). The first player is represented as intruders trying to reach a target, *i.e.*, a strategic location or an important part of the installation, and then running toward the exit. The second player is represented as the guards who have to protect the targets and, if possible, intercept the intruders. It is important to remark that none of the players knows their opponents’ movements or locations (unless a sensor alerts the guards to an intruder), but they both do their best to proceed with their goal. The uncertainty of the situation makes the problem particularly difficult. A traditional Minimax approach [22] has been chosen to handle this complexity because the players’ movements may be delimited and assumed according to the most likely places (exits, targets and so on). First, the available set of movements for the guards will be defined and the set of intruders’ movements will be supposed. Next, a function will be proposed to measure the scene’s (The scene is a specific moment in time in the scenario; in other words, a scenario in which the guards’ positions as well as the likely positions of the intruders have been set.) risk by considering the spatial relationships between the intruders and guards. This measure affects the players’ decisions, so it should embed suitable logic for generating opportune behaviours in manageable elements of the scenario, such as the guards.

In fact, some security systems in the computer networks context [26–28] use the Minimax theorem to develop defence strategies for neutralising attacks. In [28] the system is only able to detect the computer intrusion. Nevertheless, in [26,27] they are also able to generate defence strategies such as killing process or blocking IP’s. There are systems that assume a smallpox terrorist attack and use a Minimax approach to prevent it [23], and use for reasoning well known information such as the range of the attack. Others use a Minimax approach [29] for calculating the methods of guarding a territory in a grid world by intercepting the invader before he reaches the territory. In the last case, virtual territories and virtual intruders are considered and therefore the precise state of the game is always known. Finally, the authors of [25,30] also use a Minimax approach for surveillance systems but they are only able to detect faces and suspicious behaviours respectively and they do not generate any strategy to neutralise the crisis. All previous proposals in which the attack/defender scheme has been modelled by a classical Minimax approach, are not directly connected to a sensors network, hence they do not have to deal with uncertain information; furthermore, they know the precise state of the scene at each time. An exception approach, that neither works with a sensors network but deals with incomplete information, is the work described in [24] where the authors propose an interesting approach that operates in the very limited environment of a game, a context very far of our real problem and in consequence hard to use in our case.

In other words, the previous approaches provide isolated solutions to the various and specific aspects of the intrusion problem such as image detection or communications in a sensors network, but they do not tackle the problem as a whole. They do not focus on generating actions to counteract the crisis situation. The main aim of these works is to detect abnormal and independent behaviours (events) and report them to the surveillance team by generating alerts.

The aim of the proposed approach is to detect intrusions as well as determine the most appropriate neutralisation strategies. Furthermore, the proposed approach is used to solve a real complex problem, the neutralisation of intruders in strategic installations, as opposed to previous works that handle unrealistic and more academic problems. Finally, tackling the problem in its entirety, as required by a real intrusion, needs an approach capable to integrating several technologies which had not been integrated until then.

The rest of this paper is structured as follows: Section 2 outlines the Minimax algorithm and describes its application to the intrusion problem, analysing such important aspects as sensors network characteristics. Section 3 presents a performance analysis of the proposed approach in testing a set of simulations of real situations and comments on the results obtained. Finally, Section 4 summarises the principal conclusions.

## 2. Methodology

### 2.1. Context

We assume the following scenario: a strategic installation composed of one or more buildings and a location sensors network scattered throughout the main points of the infrastructure. The installation must be actively protected; that is, any unauthorised person should be detected and neutralised. For this purpose, a set of resources is available to thwart the intruders’ plans. In our context, these resources in practice are the guards, and the aim is to calculate the intruders’ next movements in order to find the best strategy for minimising the impact of the attack. There are additional elements that could also be considered as resources; alarms, doors and elevators are also concerned with threat avoidance and can be used to solve the scene intrusion. Whatever the resources, there will be only two forces: the surveillance team and the intruders. Because there are two clear sides confronting one another, we propose approaching the context as a game of two players with incomplete information, that is, with uncertainty regarding the challenger’s movements. If we consider the forces in this scenario as players, the player associated with the surveillance team will manage available resources to stop the attack, and the player associated with the intruders will try to cause the most damage. For this reason, an approach based on a game theory algorithm may be suitable.

We understand the problem as a zero-sum game, in which a participant’s gain or loss is exactly balanced by the losses or gains of the other participant(s). If the total gains of the participants are added up and the total losses are subtracted, they will total zero. Zero-sum games are frequently solved with the Minimax theorem [31–33], and this is the approach that we propose to use for our problem.

As mentioned previously, neither the intruders’ locations nor their actions are known by the guards. A set of sensors is provided throughout the scenario, and the guards are only led to the intruders’ locations when the intruders are detected. These sensors are placed close to critical points such as doors and windows and detect a presence when someone goes near them. The event and its location are notified and updated in the Minimax algorithm and is reported to the decision-support system and the surveillance team. If there are no detections, the intruders’ next locations are inferred through the Minimax algorithm. A brief description in pseudo-code of the top algorithm is shown below:

**procedure** surveillance_system(scenario, scene, depth)

  **while** (true) **do**

    events := read_sensors(scenario);

    new_scene := update_scene(scene, events);

     [best_action, best_value] := minimax(new_scene, depth, GUARDS_PLAYER);

    send_orders(best_action);

    scene := new_ scene;

  **end**

**Figure 1.**
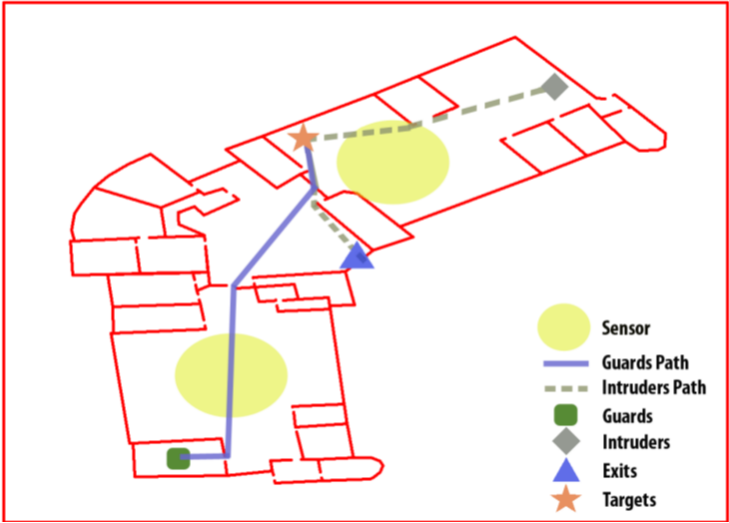
An scene with obstacle-walls and several key elements: one guard and his path to the target, one intruder and his path to the target and the exit, two sensors, one target and one exit.

Figure 1 shows an scene example in which there is one guard with his path to the intruder, one intruder with his path to the exit (after reaching a target) and two sensors in the main rooms to watch them. The lack of information is what defines the difference between a simple game and a complex, real application.

### 2.2. Sensors Network

The decision system works with information supplied by the sensors network. For our purposes, we need sensors capable of detecting suspicious events in locations the system knows well. In particular, we propose using any type of audio, video or motion sensor capable of reporting an alarm to the system. The type of sensor is not as relevant as its ability to generate an alert from a suspicious event and to connect to the system. Subsequently, it is the system that processes the information and independently decides on the nature of the sensor. A wide variety of heterogeneous sensors are suitable for inclusion in the network because the decision system evaluates the alerts and not the type of sensor. Of course, the sensor type will be important to measurement accuracy and, therefore, to the degree of belief in the detection. Because the guards watching the infrastructure can also generate events and be detected by the sensors, we have to consider a way of distinguishing between alerts triggered by the guards and those triggered by the intruders. We propose using RFID sensors [3,4,34] to identify the guards’ presence. In this way, it is possible to track their positions. Each time an alert is generated, the system will be able to recognise it as a false alarm if a RFID identification is emitted from the same place. When intruders and guards are in the same place, the system is not able to differentiate between guards and intruders because it receives the RFID identifications of the guards, as well. But in this case, guards and intruders are close enough, therefore the guards can see the intruders and inform about them. Other alternate situations are not possible in such kind of installations; since the authorized persons should carry identification (in our case a RFID card) otherwise they would be classified as unauthorized persons, *i.e.*, intruders. Furthermore the system has been designed to operate when the installations are closed; therefore we have assumed that the guards are the only authorized personnel to be there.

For the network architecture, we propose using a wireless network composed of motes. Each one of these motes is an autonomous node capable of performing a degree of processing, gathering sensory information and communicating with other nodes in the network. A similar approach can be found in [35].

**Figure 2.**
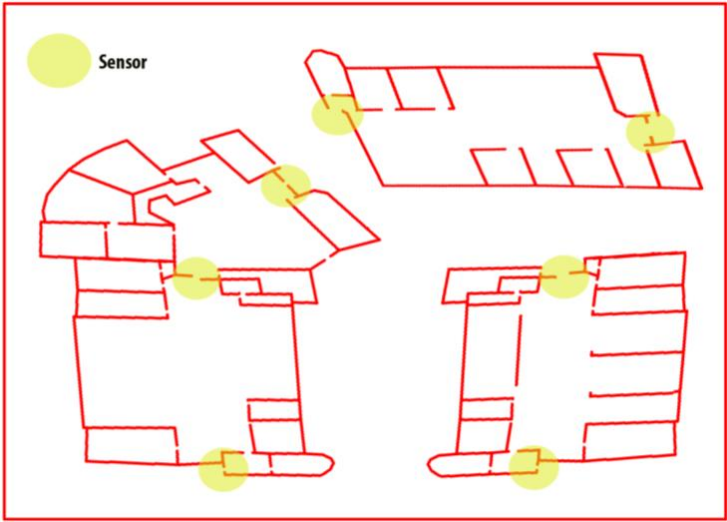
An installation map for three buildings. The locations of network sensors are shown in yellow. The sensors are placed in strategic positions, one per building exit, in an attempt to use a minimal number of sensors.

Finally, as in every surveillance system, the proper location of sensors determines the performance of the system. For this reason, in this work, the sensors have been placed at strategic points: for example, doors, exits or elevators. Figure 2 shows a possible strategy for sensor distribution in installations in three buildings. In this case, the sensors network is distributed on the main doors of the buildings.

It is important to remark that the decision system will understand every alert as a place where a suspicious activity is occurring, that is, a risk zone. Moreover the localizations of the sensors are well known during the system execution because it is assumed that the sensors are fixed in the installation in predetermined points. In this manner, practically any sensor capable of detecting a relevant event in an intrusion situation (noise, smoke, motion or lights) can be used by the reasoner, and a more scalable system is achieved. The way in which the decision system reasons from the risk zones is explained in the following sections.

### 2.3. Game Theory and Minimax Algorithm

Game theory aims to help us to understand situations in which decision makers interact. The point is to interpret interactions among decision makers as a game in the everyday sense of “competitive activities in which players contend with each other according to a set of rules” [20]. As in other sciences, game theory consists of a collection of models. The present approach focuses in particular on models inspired by the theory of rational choice. Thus, players choose the strategy that best maximises their possibility of winning from among all the available strategies.

The approach of game theory that fits best with the intrusion context is the Minimax algorithm, which is based on the Minimax theorem shown below.

**Minimax Theorem:** *For every two-person, zero-sum game with finite strategies, there exists a value V and a mixed strategy for each player, such that (a) given player two’s strategy, the best pay-off possible for player one is V and (b) given player one’s strategy, the best pay-off possible for player two is −V.*

In our context, we understand the first player as the force trying to protect the strategic installation, the guards, and the second player as the force attempting to attack the infrastructure, the intruders. Each player will search among all the possible next actions for the one that will maximise his gain and minimise his loss. This strategy also implies minimising the challenger’s gains and maximising his losses. If we consider these actions to be the correct moves for the game, the Minimax algorithm with alternate moves can be used to select the best next move, that is, the best action. To work properly, the Minimax requires numerically measuring how well the game board is set (in our context, this would be the state of the scenario, *i.e.*, the scene). Every time a new action is executed, a new state of the scenario is generated; it is possible to distinguish the best actions by scoring the old and the new states. The first player will prefer the states with higher scores, and the second player will prefer the states with lower values. Thus, the first player is called the maximising player, and the second player is called the minimising player, the name of the theorem is *Minimax*.

If the process is repeated, from these new states and the execution of new actions, additional future and advanced states can be generated. The idea is to repeat the procedure until a winning strategy is discovered that is impossible for the other player to avoid (because none of his moves will help him avoid defeat). The first player selects the move that maximises the minimum value of the position resulting from the opponent’s available next moves. In other words, player one chooses the move that avoids his opponent’s best move by considering the moves that would become available to the other player as a result of each of his own possible moves. The positive and negative infinite values are assigned to the winner states for the maximising and minimising player, respectively; however, this process is generally only possible at the very end of the game because it is not computationally feasible to look ahead as far as the completion of the game. Instead of these extreme values, finite scores are given as estimations of the degree of belief that they will lead to a win for one player or the other. These estimations of the goodness of a state, that is, how favourable it is for the intentions of the player, are supplied by an estimation function, which considers all of the possible following sequences in order to estimate the goodness of the state.

A pseudo-code description of the Minimax algorithm is shown in the next lines:

**function** [best_operator, value] := minimax(state, depth, player)

**if** depth <= 0 **then**

  **return** [no_action, heuristic_estimation(state, player)];

**else**

  best_operator := no_action;

  **if** maximizing_player(player) **then**

    value := -Infinite;

  **else**

    value := +Infinite;

  **end**

  **for** op ∈ operators(player) **do**

    new_state: = apply_operator(state, op);

    [last_op, last_value] := minimax(new_state, depth – 1, ¬player)

    **if** maximizing_player(player) **&&** last_value > **value**

      value := last_value;

      best_operator := last_op;

    **else if** ¬maximizing_player(player) && last_value < value **then**

      value := last_value;

      best_operator := last_op;

    **end**

  **end**

**end**

**return** [best_operator, value];

In the following sections, the available set of actions for each player in the proposed system will be discussed as well as the measure function used to evaluate the state of the game.

### 2.4. Operators

In our context, a game state is represented by the map of the protected space (the map of the building) and the set of key elements. We understand any object, place or event that modifies the Minimax value measured by the evaluation function as key elements; in this case, elements that have an influence on the development of the intrusion. The key elements defined in this paper are the following:

- Guards: the guards’ location is a very important parameter. The positions of the guards strongly determine the risk of the intrusion scene. Likewise, the number of guards must be taken into account when estimating the risk.
- Intruders: in the same manner, the number of intruders and their locations must be considered in the risk estimation of the scene.
- Targets: there are objects and places of particular relevance and high probability of being the intruders’ objective, so they must be considered for special protection and, therefore, as part of the risk estimation.
- Exits: the exits indicate places to leave the scenario, that is, the main points of escape from the guards’ space.
- Events: they represent the alerts emitted by the sensors network. Every time a new alert is generated, the system is notified, and this information is added to its knowledge base. The position covered where the alert was generated must be evaluated as a risk zone and should be examined by the guards.

It is important to remark that in practice, the intruders’ locations and their intentions are not known. This is the main difference with respect to a game approach. The guards do not know what the intruders are doing, only their approximate locations when they are detected and an event is signalled. This statement generates a high level of uncertainty and greatly increases the complexity of the system task.

In practice, for player one (who tries to neutralise the intrusion), every location other than the guards’ positions is considered to be a risk zone and must be reached and inspected to prevent and capture the intruders. In other words, the intruders’ locations, targets, exits and event locations are risk points that should be considered to be the main zones to be inspected by the guards. Each of these risk zones has a risk level according to its relevance in the scenario. The more relevant a place or an element, the higher the risk level is. These places are of two kinds: (1) risk zones created according to the knowledge of the expert in charge of the building surveillance as well as according to the security policy (structural risk zones) and defined prior to the start of the system execution; and (2) risk zones that appear in the scenario during a crisis (dynamic risk zones). Every sensor activation characterised as abnormal by the system will be added to the scenario as a new risk zone to be explored. Likewise, as explained in the risk estimation section, a dynamic risk zone can be removed if no more abnormal activity is detected during a defined time interval.

The risk of the current scene is calculated by a function (see Section 2.6) that obtains an assessment of the game state for both players, that is, how favourable the scene is to their interests. Therefore the scene’s risk has to increase with the distances between the guards and the risk zones. Consequently, one of the main actions that player one can execute is move the guards to the risk zones. Since each guard goes to a risk zone, there are a number of possible ways to approximate this behaviour, and it is here that the Minimax algorithm makes sense because it is able to evaluate the possible combinations of present and future actions, both the first player’s moves and the second player’s reactions. For the second player (intruder), the system always assumes that the main actions or moves will be to go to the targets or the exits. These two behaviours cover their probable intentions, that is, reaching a strategic place and escaping.

However, moving a guard should not be the only possible action in order to change a scene for reaching a new state or new scene, indeed actions may be accomplished over any operable element of the scenario such as doors, windows or alarms, and so on. Note that in the case of the intrusion force (player two), the only operable elements are the intruders that may be moved to a target or to an exit. Moreover, the generation of new states or scenes is accomplished by the combination of the actions that may be realized over the operable elements, understanding that only one action can be accomplished over the same element at each turn. For this reason in the search process of the best strategy, given a particular scene the proposed approach considers more than one action per turn, which highly increases the computational complexity of the problem. Consequently the search must be bounded and therefore the node expansion and exploration may progress only until a specified depth level of the Minimax tree. When the node expansion process has finished, the risks of the scenes (states) in the deepest level explored are assessed and values are propagated across nodes in the tree until the start state (root node) as explained in Section 2.3. In this way, given a scene, it is possible to decide the best set of actions (neutralization strategy) to be performed at each turn.

Table 1 shows the operable elements considered in this paper and the set of possible actions over them.

Alarms, doors as well as guards will be led by the first player (the guards’ team); on the other hand, the second player only will lead the intruders’ movements. The closing (or the opening) of a door can increase (reduce) the distance between two key elements on the scene, therefore these elements can be a very useful resource for the guards’ team. The intruders cannot activate the doors because doors are remotely controlled by the surveillance system and consequently the intruders do not have access to the same. The alarms are also been considered because they can warn the staff located on the crisis place to make them aware of the situation, therefore, they only make sense for the guard’s team. Furthermore alarms can only be operated by the surveillance system.

Since each strategy allows several actions, then a strategy can be formally characterized by Equation (1):

(1) $$\mathrm{strategy}=(a_{1},\cdots ,a_{n})|a_{i}\in A\wedge i\in \mathrm{OE}$$

where *A* is the set of possible actions *a_i_* for the operable element *i* and *OE* is the set of all operable elements. It should be noticed that *OE* is not the same set for both players (Table 1). According to the player, the strategies will be composed by a different number of actions. For example, if the scenario has *n_g_* guards, *n_d_* doors and *n_a_* alarms, then any strategy applied by the first player (the guards team) might contain *n_g_* + *n_d_* + *n_a_* actions, on the other hand, if the scenario has *n_i_* intruders then any intruder strategy might contain *n_i_* actions. Therefore, the strategies are the operators that shall be applied during the search in order to generate a new state and therefore the states’ number shall be very high. Indeed, it is such a high number of possible new states (scenes) that the exploration strategy adopted so far may not be enough to handle all nodes that might be generated since search space really increases exponentially. In fact, if we consider *n_rz_* risk zones, *n_g_* guards, *n_i_* intruders, *n_d_* doors, *n_p_* risk zones of type exit or target and *n_a_* alarms, then in each new level, the algorithm will have to evaluate (1 + n_rz_)^n_g_^ 2^n_d_^ 2^n_a_^ new states in the case of the guards turn or (1 + n_p_)^n_i_^ new states in the case of the intruders turns. Therefore, it is imperative to combine a pruning strategy with the previous depth-limitation method in order to reduce the exploration time and in consequence the system time response.

### 2.5. Alpha-Beta Pruning

To reduce the number of states evaluated in the tree generated by the Minimax search, an Alpha-Beta pruning [36] has been included in the algorithm. The Minimax search is a depth-first search, hence a node can have an estimation value without having explored all its children. If for example, we are in a maximizing node with an estimation value *v_1_* obtained by recursion through its first child, then, in its following children, if a value *v_2_* lower than *v_1_* is achieved, it is not necessary to expand more branches in any of the remaining children because *v_2_* can only decrease (thus we are now in a minimizing level) and the maximizing node will never choose it.

At each time, the algorithm maintains two values, alpha and beta, which represent the minimum score that the maximizing player has achieved so far and the maximum score that the minimizing player has got respectively. Initially, alpha is negative infinity and beta is positive infinity. As the recursion progresses the “window” becomes smaller. When beta becomes less than alpha, it means that the current node value can only decrease (thus alpha is greater) and then there is no need to explore further nodes. The process explained is captured in the pseudo-code below.

**function** [best_operator, value] := minimax_ alphabeta (state, depth, player, alpha, beta)

**if** (depth <= 0) **then**

  **return** [no_action, heuristic_estimation(state, player)];

**else**

  best_operator := no_action;

  **if** maximizing_player(player) **then**

      value := −Infinite;

  **else**

      value := +Infinite;

  **end**

  **for** op **∈** operators(player) **do**

    **if** (alpha < beta) **then**

      new_state := apply_operator(state, op);

      [last_op, last_value] := minimax_alphabeta(new_state, depth – 1, ¬player, alpha, beta)

      **if** (maximizing_player(player) **&&** last_value > value) **then**

        value := last_value;

        best_operator := last_op;

        beta := last_value;

      **else if** (¬maximizing_player(player) && last_value < value) **then**

        value := last_value;

        best_operator := last_op;

        alpha := last_value;

      **end**

    **end**

  **end**

**end**

**return** [best_operator, value];

Finally, if b is the mean node children number of the Minimax search tree, the Alpha-Beta pruning on average can reduce the number of children of the tree approximating $O(\sqrt{b})$ [36].

When the alpha-beta pruning is used, the invocation to the Minimax algorithm must be replaced by the Minimax with the alpha-beta pruning (the previous pseudo-code) in the top algorithm, *i.e.*, the pseudo-code of Section 2.1.

In the next section, we will explain how to estimate the risk of a scene. We use a heuristic function to determine how favourable the scene is for each player. The aim is to measure the scenario state to ultimately perform the best action in order to neutralise the intrusion.

### 2.6. Risk Estimation Function or Entropy

As described in Section 2.4, the two forces, both guards and intruders (players), make their moves with the aim of generating scenario states that are more favourable to their interests. Therefore, it is necessary to define a function that estimates the risk of the current scene, obtaining an approximate evaluation of the game state for both players. The designed function is called “entropy” because it measures the disorganisation level of the scene. The more disorganised a scene is, the more favourable it is for an intruder. The disorganisation is directly related to the distances among the different risk zones. For example, disorganisation increases as an intruder nears a target or an exit and decreases when a guard is close to an intruder (It should be noted that the risk zones in a scenario are managed according to the events reported to the decision system and the initial state of the scene. As we explained previously, each time that a sensor issues an alert, a new risk zone is added unless it is identified as a guard event. The exits and main possible objectives are inserted as risk zones into the initial state of the system.). To measure the distances over a scenario with obstacles, the decision system uses a path planner based on a technique known as Delaunay Triangulation [37]. This technique divides the space into triangles. The sides of these triangles can be labelled as constraint or unconstraint. The constraints ones are used to represent obstacle edges. Finally, the path finding algorithm implement a TA* search over these triangles (similar to the A* algorithm but adapted for triangle nodes), jumping among them only through the unconstraint edges. The main point of this technique is its capability to support obstacles.

When implementing the risk estimation, the values are bounded by +∞ and zero. If the state is a winner for the player one (guards), it assigns the lowest value possible, zero. Otherwise, if the state is a winner for the intruders (player two), it assigns the highest value possible, +∞, which is the maximum disorganisation. In other words, if the value is closer to zero, the situation is more favourable to the guards; otherwise, it is more favourable to the intruders. Therefore the first player (the guards’ team) is the player who tries to minimise the value of the function as opposed to the second player (the intruders’ team) who tries to maximise the value of the function. It should be noted that the strategy proposed is not the common way of using minimax algorithm and the alpha-beta pruning in a game. Max nodes are generally nodes where the computer (guards in this case) is making decisions, so they try to maximize the value of the evaluation function. However in the problem tackled the objective is to reduce the distances between guards and intruders. Therefore a function directly dependent on the distance has been defined and in consequence, the objective is to try to minimize the value of the evaluation function. This change in the target for the function does not alter the logic neither the behaviour of the minimax algorithm and the alpha-beta pruning.

An approach based on the distances between scenario elements is proposed for measuring different scenes according to risk zones. In fact, entropy is defined to be directly proportional to the sum of distances. Moreover, taking the relevance of the elements in the intrusion scene into account, each element is assigned an adjustable factor risk that can reduce or increase in weight in the scene according to its importance as a risk zone. The value of the risk factor varies between 0 and 1. This risk factor parameter will multiply the distance values between a guard and its associated element, *i.e.*, the risk zone. In this way, the entropy will penalise the risk zones with a low risk factor (less important) and will give priority to the more important risk zones (those with a high risk factor). In addition, each risk zone has a timer that is used to reduce the risk factor. After a certain amount of time with no alerts issued in the same risk zone, the risk decreases; this decrease is achieved by readjusting the risk factor. The time needed to elapse to reduce the risk factor has to be set according to the knowledge of the security expert as well as the installations’ security policy. Of course, these changes will strongly affect the behaviour of the system.

In summary, the idea of entropy is used to measure the risk of a scene according to the distances between the risk zones. Equation (2) shows the proposed entropy function:

(2) $$\mathrm{entropy}(\mathrm{state})=\{\begin{array}{lll} \sum_{i=1}^{n_{G}}\sum_{j=1}^{n_{Z}}\mathrm{distances}(G_{i},Z_{j})\cdot \mathrm{riskfactor}(Z_{j}) & \mathrm{for} & \mathrm{Guards}\\ \sum_{i=1}^{n_{I}}\sum_{j=1}^{n_{T}}\mathrm{MAXENTROPY}-\frac{\mathrm{distances}(I_{i},T_{j})}{\mathrm{riskfactor}(T_{j})} & \mathrm{for} & \mathrm{Intruders} \end{array}$$

where *distances*(*G_i_, Z_j_*) is the distance between the guard *i* and the risk zone *j*, *n_G_* is the total number of guards, *n_Z_* is the total number of risk zones, *distances*(*I_i_, T_j_*) is the distance between the intruder *i* and the risk zone of exit type or target *j*, *n_I_* is the total number of intruders and *n_T_* is the total number of risk zones of exit type or target. Finally, *riskfactor*(*T_j_*) retrieves the risk factor of the risk zone exit or target *j*. We assume the intruders are going to do their best to reach the exits and the targets. In consequence, only the distances between intruders and these zones (exits or targets) must be considered. Moreover, the same function is used for both players, but always regarding the different behaviours associated to each force. This is not the common way to define the evaluation function in games because habitually players have the same set of actions to be selected and executed and this is not the case in the problem tackled. For example, the intruders do not know where the guards are and on the other hand the guards do know where suspicious events are happening. For this reason, concerning the guards, the distance between them and the risk zones is used, and concerning the intruders, this distance cannot be used because they do not know where the guards are. Moreover actions to be accomplished by guards are different of those to be realized by the intruders.

According to the entropy, that is, to the risk estimation function for the scene, the Minimax algorithm selects the best set of actions to solve the intrusion scenario. The entropy is the main parameter used to change the behaviour of the system because the system will make the best decisions in accordance with the entropy. For example, if we want guards to specially protect the exits, the entropy may be changed to add a higher risk factor to these key elements.

Finally, it is important to note that this approach could represent any type of crisis. By interpreting any dangerous level as a risk zone, any crisis scenario can be modelled and measured by the proposed evaluation function. The decision system will choose actions according to the retrieved value and independent of the type of risk, so the overall system can easily be adapted for any type of attack.

## 3. Results

### Final State Result

**Figure 3.**
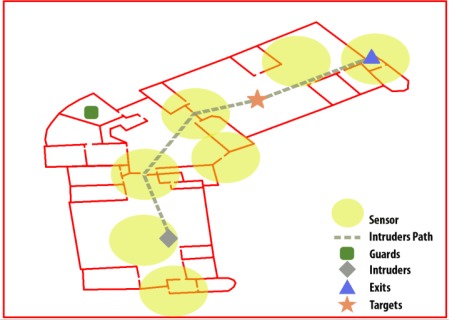
The simulation of a real intrusion situation with 1 guard, 1 intruder, 1 target and 1 exit.

The setting of a complex building has been chosen to evaluate the developed system. Four types of key elements have been selected: guards, intruders, exits and targets, as shown in Figure 2. In this first attempt, the doors or the alarms are not considered in order to clarify the tests and only the operable elements which have the most complex behaviour (the guards) have been taken into account for neutralising the intrusion. The simplification makes the results easier to be analyzed without invalidating the test method used. In fact the method followed allows analysing easily if the guards show a rational behaviour in the neutralization strategies generated by the system. The locations of the guards, intruders and targets were determined randomly in the space. The exits were also randomly generated but were always in the position of any of the building’s main doors. The number of elements can change from 1 to 2. The Minimax algorithm will be run, and the guards’ locations in the scenes will be updated according to the best moves predicted by the algorithm. The guards should catch the intruders as soon as possible; the intruders will try to reach a target and then leave the building through any exit. Specifically, the intruders will select a random target from the available ones and the closest exit to it. They will advance until they reach the target and then change their objectives, moving toward the exit. The guards will try to intercept the intruders as quickly as possible. The intruders’ starting point is known by the guards, and a set of sensors is distributed throughout the scenario. If one of the sensors is activated by an intruder, the intruder’s location is updated in the Minimax algorithm and made accessible to the guards. The intruders are inserted with a factor risk of 1 (the highest), and the exits and targets with a lower factor, 0.5, so the entropy will send the guards to the intruders rather than to the exits or targets. The scenario is completed with the sensors network. As shown in Figure 3, there are 7 sensors placed in critical points: 3 are covering the exits (one for each exit), 2 are covering the doors that connect the main rooms and the remaining 2 are covering the space in the larger rooms. The sensors can differentiate between the intruders and guards. The guards have an RFID tag, so if a presence is detected and there is no RFID identification, an intruder presence is assumed.

We ran the system for 100 randomly generated intrusion scenes. When the final scenario state was reached, we analysed the results regarding the intruders. There are three possible basic outcomes: the intruders were captured before arriving at the target, they were captured after arriving at the target but before arriving at the exit or they reached the exit and escaped.

Table 2 shows several configurations of guards, intruders, exits and targets. For each configuration, 100 simulations of real situations have been executed. The situations have been designed following the requirements and security policy established in the HESPERIA project [18]. This project involved security experts who designed the real-life intrusion situations.

The final state of the simulations is divided into three categories according to the final state of the intruders: (a) captured before reaching a target; (b) captured after reaching a target or (c) not captured. The percentages expressed are for the 100 simulations. For example, for the configuration (1, 2, 2, 2), that is, number of guards = 1, number of intruders = 2, number of exits = 2 and number of targets = 2, the intruders escaped in 25% of the cases, which is nearly the worst percentage obtained. The configuration (1, 1, 2, 2) was also adverse, with 27% of cases allowing intruders to escape. The best results were obtained for (2, 2, 1, 2). Although there were two intruders, there were also two guards and only one exit. Only 3.5% escaped. Finally the maximum depth for the search process was set to 4. A higher number would have increased hugely the response time because the computer in charge of executing the simulations was a Pentium IV 1.500 MHz with 1 GB Ram memory. Depending on the computer resources this parameter should be adjusted. For each one of these tests, the average number of decisions taken was 15, since 100 experiments were executed and the total time to accomplish them was 4.5 h, therefore it took (4.5·3600)/(15·100) = 10.8 s to make a decision. Considering the computer used, a Pentium IV 1.500 MHz with 1 GB Ram memory, it can be concluded that the system is suitable for giving a response in time.

Table 3 shows that according to the different sets of start scenes, randomly generated, for a scenario as one represented in Figure 3 (explain above), the intruders were caught before arriving at their target in 59.25% of the occasions and after they had reached their target in 27.25% of situations. In only 13.5% of cases were the intruders able to exit. In the simulations, an intruder is considered captured if a guard comes close enough to him. In our case, the distance between them has only to be a few metres, the distance covered by a person in 2 seconds. If the path between the guard and the intruders is a straight line (they reach to see each other), the capture distance is doubled. An intruder achieves escape when he reaches an exit.

Concerning the behaviour observed in the final strategy from the point of view of a person, the guards showed a rational behaviour. In most cases they selected a path that intercepted the intruders or they decided to go to an exit when the targets were protected or go to a target when the target was in danger. In few cases, they selected the wrong exit or target while the intruder ran out through another side. This was mostly due to the presence of two possible exits (or targets) equidistant from the intruder. Finding the appropriate strategy in this situation is difficult even for a human.

Before finishing this section it is important to note that trials have been carried out with a not very fast computer. Therefore, it is still possible to use a more sophisticated and fast equipment for computation, for example a computer based in GPUs, a cluster or even a grid of computers, if necessary. Despite the response time can be increased by faster computation equipment it will be always a limit for response time because it increases exponentially with the number of guards. At this point it is important to realize the context in which we are at the moment, the surveillance of an installation. In this context the number of guards required to protect a zone is proportional to the size of space to be protected. Furthermore, in real cases, a space must be split into zones that can be efficiently surveyed by a fix number of guards, because in real situations the important question is how long it takes a guard to reach the farthest point from him, so there is a physical limit that is more significant than the computational one. Therefore, in general, the space to be surveyed shall be split in *m* zones with a size that ensure the efficient protection of each of them by a fix number of guards (*n*). In consequence the complexity of the problem equals *O*(*m·g*(*n*)), where *g*(*n*) is the exponential-time part but in the surveillance context n can be adjusted to the response time requirements of the system. In fact, in real situations guards work in couples, surveying spaces with a size and complexity similar to those used in the trials presented.

## 4. Conclusions

There is little doubt that the current market is in need of integral solutions to enhance the security and control of particularly sensitive public infrastructures, such as electrical substations, water tanks or telecommunications stations, or large public spaces such as airports, train stations or city centres. It is especially important to provide smart technology that reduces human error, a critical advantage in this type of location. Until now, the analysis and decision making was the responsibility of a surveillance team that was required to process all of the information received in a short time in order to make suitable decisions oriented to minimising the impact of the attack as quickly as possible. Therefore, the success of the final action largely depends on the speed and accuracy with which the decisions are made. Thus, it is imperative to design and develop decision-making systems for automating the decision generation process from data supplied by a sensors network, which will reduce the time for decision generation as well. However, the large number of variables required (e.g., distances, number of available resources, number of places attacked and blocked doors) to adequately solve crisis situations makes the decision generation problem complex and non-trivial to solve.

In this context, the system that we have described makes decisions concerning several complex scenarios in real time, *i.e.*, it chooses the best action among all possible actions and takes the most relevant features of the attack into account. The proposed approach is based on a Minimax algorithm combined with a Alpha-Beta Pruning method for selecting the best behaviour among all of the available strategies of neutralisation, considering these strategies as a set of actions across the elements of the scene: for example, moving a guard, blocking a door or turning on an alarm. The distances between the different scenario elements have been used to measure the risk of the scene (entropy function), so a path planning technique has been integrated to reach a high-quality performance.

The most important features of the proposal can be summarised in the following points:

- One action per independent element at each time. The system retrieves as many actions as there are independent resources in the scene. In our case, each guard is given a move to be performed so that complex defence strategies can be generated.
- No training process is needed. There is no need to train the system with an initial set of training intrusions as required by other existing systems.
- A large number of sensors can be used regardless of their type of perception. A sensor’s ability to alert the decision support system is what matters.
- Encapsulated logic in the entropy function. The behaviour of the decision system can completely change by modifying the function of risk estimation.
- The system can easily be adapted to represent any type of attack. With the methodology of adding/removing risk zones, the system evaluates a risk and not what causes it, building, in this manner, a modular and scalable reasoning.
- Accurate and scalable representation of an intrusion scene. The scene is properly represented and supported, and if there are new key elements for evaluation of the scene, they can be added easily. The logic of the entropy function determines where they should be analysed and properly considered.
- The intrusion scene can be evaluated at a particular depth chosen by the user according to the level of study supported or required. The Minimax algorithm can make the searches more or less exhaustive to correspond with the specified depth. This means that for complex environments, computers will require architectures with high processing capabilities.
- The risk factors for each type of event can be adjusted to focus the relevance of the scene to the risk zones with higher values.

To guarantee the performance of the application, this study has been conducted using a real infrastructure, and the testing has been performed against a battery of simulated real situations that can be completed in the infrastructure. The results show that in 86.5% of the cases, the system achieved capture of the intruders, and in 59.25%, the intruders were intercepted before they reached their objective.

Finally, future works are required for setting up the methodology to be followed for obtaining the best distribution of sensors, keeping in mind the features of the proposed system. Given an installation, the aim is to find the sensor locations that maximise intruder captures. Furthermore, sensor measurements are inherently uncertain and often inconsistent. Therefore an appropriate consideration of uncertainty and identification/elimination of inconsistent measurements is required and in consequence it will be part of the future works, even though the already developed system can handle properly part of the sensors’ uncertainty through its reasoning based in risk zones. Among possible strategies for solving uncertainty and inconsistency in data, data fusion from multiple sources is especially interesting. The idea is to replicate sensors in such a way that at least the most conflictive zones are being covered for more than one sensor of the same or different kind.

Moreover, the current set of operators could be increased to support more complex types of actions. For example, actions such as closing or blocking doors and elevators, turning on the alarm or using an object can be added to provide the system with new methods of neutralising an intrusion. New elements such as stairs, elevators, alarms, windows and fire extinguishers could also be added to the scenario and used by the system to solve the crisis.

## Figures and Tables

**Table 1.** Operable elements per player and the set of actions over them.

**Player**	**Operable elements**	***Actions***
First (guards’ team)	guards	*no_action, move_to(rz) | rz is a riskzone*
	doors	*open, close*
	alarms	*turn_off, turn_on*

Second (intruders’ team)	intruders	*no_action, move_to(p) | p is an exit or target*

**Table 2.** Results of the test that simulated the system behaviour beginning from different starting configurations (*Guards, Intruders, Exits, Targets*).

**(Guards, Intruders)**	**(Exits, Targets)**				
	**(1, 1)**	**(1, 2)**	**(2,2)**	**(2,1)**	**Final State**
(1, 1)	45%	56%	46%	45%	Captured Before Target
	34%	29%	27%	39%	Captured Before Exit
	21%	15%	27%	16%	Not Captured

(1, 2)	50%	53%	50.5%	48.5%	Captured Before Target
	32%	30%	24.5%	27%	Captured Before Exit
	18%	17%	25%	24.5%	Not Captured

(2, 2)	68%	73.5%	67.5%	72%	Captured Before Target
	28%	23%	25.5%	20%	Captured Before Exit
	4%	3.5%	7%	8%	Not Captured

(2, 1)	72%	74%	59%	68%	Captured Before Target
	24%	18%	31%	24%	Captured Before Exit
	4%	8%	10%	8%	Not Captured

**Table 3.** Percentages of different states of the intruders after the testing battery.

**Final State**	**%**
Captured Before Target	59.25%
Captured Before Exit	27.25%
Not Captured	13.5%
